# Spatiotemporal Regulation of FMNL2 by N-Terminal Myristoylation and C-Terminal Phosphorylation Drives Rapid Filopodia Formation

**DOI:** 10.3390/biom13030548

**Published:** 2023-03-17

**Authors:** Lina Lorenzen, Dennis Frank, Carsten Schwan, Robert Grosse

**Affiliations:** 1 Institute of Experimental and Clinical Pharmacology and Toxicology, Medical Faculty, University of Freiburg, 79104 Freiburg, Germany; 2 Centre for Integrative Biological Signalling Studies—CIBSS, 79104 Freiburg, Germany

**Keywords:** actin, formins, FMNL2, filopodia, protein kinase C

## Abstract

The actin nucleating and polymerizing formin-like 2 (FMNL2) is upregulated in several cancers and has been shown to play important roles in cell migration, invasion, cell–cell adhesion and filopodia formation. Here, using structured illumination microscopy we show that FMNL2 promotes rapid and highly dynamic filopodia formation in epithelial cells while remaining on the tip of the growing filopodia. This filopodia tip localization depends fully on its N-terminal myristoylation. We further show that FMNL2-dependent filopodia formation requires its serine 1072 phosphorylation within the diaphanous-autoregulatory domain (DAD) by protein kinase C (PKC) α. Consistent with this, filopodia formation depends on PKC activity and PKCα localizes to the base of growing filopodia. Thus, a PKCα–FMNL2 signaling module spatiotemporally controls dynamic filopodia formation.

## 1. Introduction

The actin cytoskeleton plays a critical role in regulating various cellular functions, such as migration, adhesion and alterations in cell morphology, through its remodeling [1]. One group of actin regulators is comprised of formins. Formins are a conserved family of actin regulators defined by the presence of a formin homology domain 2 (FH2), which is crucial for their ability to nucleate and elongate linear actin filaments [2]. Unlike many other actin nucleators, such as the Arp2/3 complex and Spire, formins remain associated with the barbed ends during filament elongation [3,4]. Moreover, the antagonization of capping proteins makes formins well-suited for efficiently generating long actin filaments. Among the formins, the Diaphanous-Related Formins (DRFs) are the best characterized subfamily. The formin-like proteins FMNL1, 2 and 3 belong to this DRF subfamily [5]. FMNL2, also known as FRL3 or FHOD2, regulates actin polymerization using its functionally conserved formin homology 1 (FH1) and FH2 domains [2,5]. Similar to other DRFs, the actin nucleating activity of FMNL2 is regulated by autoinhibition. This autoinhibition is facilitated through intramolecular interactions between the C- and N-terminus [6]. Binding of Rho-GTPases to the GTPase binding domain, as well as post-translational modifications, such as phosphorylation at different sites, can influence the autoinhibited state and initiate the actin polymerizing activity of FMNL2 [7,8,9,10]. In a previous study, we identified serine 1072 in FMNL2 as a target site of PKCα phosphorylation, releasing FMNL2 from its autoinhibited state [10].

FMNL2 largely localizes at protrusive structures, such as filopodia and lamellipodia [11,12]. Thus, recent studies identified FMNL2 and also FMNL3 as important drivers of filopodia formation [11,13,14,15,16]. Filopodia are finger-like protrusions that extend from the plasma membrane, with a bundle of actin filaments as their key structural component. The actin filaments in this bundle are arranged in parallel, with their barbed ends pointing towards the filopodial tip. In the dense tips of filopodia, numerous proteins have been observed, including integrins [17]. In addition to formins, the extension of filopodia is subject to regulation by other actin regulators, including Ena/Vasp, and by actin-capping proteins. Actin-bundling proteins, for example fascin, alpha-actinin or villin, are responsible for the packing of filaments. Additionally, the transport along the actin filaments of filopodia is facilitated by motor proteins, such as myosin-X [17]. Filopodia have a variety of cellular functions, including cell migration and adhesion while probing the cellular environment [18]. Filopodia and filopodia-like structures have been shown to be involved in cancer cell invasion. In ovarian cancer cells, a positive correlation has been established between an elevated number of filopodia-like structures and invasiveness, thereby indicating the potential role of such structures in mediating the metastatic dissemination of malignant cells [19]. Various proteins, that are involved in filopodial formation and function, such as fascin, have been linked to cancer progression and metastasis. Analysis of a set of filopodia-related genes suggests that breast carcinomas with an unfavorable prognosis exhibit an upregulation of genes associated with filopodia [17]. In addition to facilitating the metastasis of cancer cells, filopodia-like protrusions seem to play a pivotal role in promoting survival and proliferation of the cancerous cells apart from the primary tumor [19].

FMNL2 also plays an important role in cell migration [11] and is involved in metastasis of several cancers, such as colorectal cancer where it is associated with aggressive tumor development, hepatocellular carcinoma and gastric cancer [9,20,21,22,23]. In melanoma cells, FMNL2 is recruited to the leading edge of migrating cells and promotes lamellipodial extension by elongation of Arp2/3 complex–nucleated filaments [11].

FMNL2 can undergo post translational N-myristoylation which is necessary for its recruitment to the plasma membrane and other intracellular membrane compartments [10,24,25,26]. This posttranslational N-myristoylation was previously implicated in the formation of filopodia [13]. Structural studies suggest that FMNL2 itself could induce negative membrane curvature [27]. Additionally, FMNL2 recruits I-BAR proteins to the site of filopodial assembly which cooperatively aid in filopodia formation by their membrane-bending and actin-binding capabilities [28].

In this study, we analyze the phosphorylation-dependent regulation of filopodia elongation by FMNL2. SIM microscopy in living cells revealed that FMNL2 directly polymerizes actin at the tip of filopodia. The processive elongation of filopodia was strongly dependent on phosphorylation of the serine residue at position 1072 in FMNL2 by the activity of PKC.

## 2. Materials and Methods

### 2.1. Cell Lines, DNA

MCF10A cells were cultured at 37 °C with a 5% CO_2_ atmosphere in DMEM/F12 (Gibco Life Technologies, Paisley, UK) supplemented with 5% horse serum, 20 ng/mL epidermal growth factor, 10 µg/mL insulin, 0.5 µg/mL hydrocortisone, 100 ng/mL cholera toxin, 100 U/mL penicillin and 100 µg/mL streptomycin. The MCF10A and HEK293T cell lines were acquired from ATCC: MCF10A, #CRL-10317; HEK293T, #CRL-3216. MCF10A FMNL2-KO cells were generated by CRISPR/Cas-mediated gene deletion [16]. All cell lines were tested monthly for mycoplasma contamination.

MCF10A stable cell lines were generated by lentiviral transduction. Viruses were produced by transfection of HEK293T cells with Lipofectamine 2000 according to the manufacturer’s instructions with packaging (psPAX2), envelope (pMD2G) and expression plasmids: PWPXL-FMNL2-GFP (Wang et al., 2015 10) and PWPXL-FMNL2-S1072A-GFP (Wang et al., 2015 10). Supernatants were harvested 48 h after transfection and filtered through a 0.45 μm filter. MCF10A cells were infected with the virus supernatant and 48 h after infection, the cells were trypsinized and passaged. Cells were FACS-sorted to maintain a homogeneously expressing population of cells. 

### 2.2. Reagents, Plasmids

Cell culture reagents were purchased from Invitrogen. CK-666 (100 µM, Sigma, St. Louis, MO, USA) and BIM (2 µM, Enzo Life Sciences, Villeurbanne, France) were used for drug treatments.

For transient transfections, GFP-FMNL2 [11], GFP-PKCα [10] and actin-chromobody [29] SNAP plasmids were used.

The GFP-FMNL2 plasmid was a gift from Dr. Klemens Rottner.

### 2.3. Western Blot

Proteins were separated by sodium dodecyl sulfate polyacrylamide gel electrophoresis (SDS-PAGE) and transferred to nitrocellulose membranes. The membranes were blocked for 1 h at room temperature and then incubated with primary antibodies diluted in blocking buffer on a shaker for 1 to 2 h at room temperature or overnight at 4 °C. The membranes were incubated with horseradish peroxidase-labeled secondary antibody for 1 h. Ultrasensitive enhanced chemiluminescence (ECL) substrate was used for protein detection. Primary antibodies used were anti-FMNL2 (1:250, mouse monoclonal, SCB, Heidelberg, Germany) and anti-Tubulin (1:1000, rabbit monoclonal, CST, Leiden, The Netherlands). Secondary antibodies used were anti-rabbit IgG-HRP (1:5000, goat, Biorad, Feldenkirchen, Germany) and anti–mouse IgG-HRP (1:5000, goat, GE Healthcare, Chicago, IL, USA).

### 2.4. Microscopy, Live Imaging and Image Analysis

MCF10A cells were seeded into 35 mm glass bottom dishes (Greiner, Frickenhausen, Germany). The cells were subjected to live imaging at least 24 h after transfection using Lipofectamine 3000 according to the manufacturer’s instructions.

SNAP-Cell 647 SiR (1:2000, NEB, S9192S) was added, followed by a 30 min incubation before imaging. Structured illumination microscopy (SIM) imaging was performed with an ELYRA 7 microscope (Zeiss, Oberkochen, Germany) equipped with a 63  ×  1.4 Oil DIC objective and a Pecon incubation chamber, providing a stable environment for the samples at 37 °C and 5% CO_2_. All acquired imaging data were SIM processed using Zen 3.0 black edition. For SIM reconstruction, the “Standard” end criterion of the manufacturer was applied. The “baseline-shift” method was used and the threshold was set as the first peak in the histogram for representative images. Time lapse images were acquired as a series of single-plane images with the minimal time interval and further processed using sliding processing (Burst-mode, Zeiss). Images were further analyzed with Imaris 9.9.0 and FIJI-ImageJ 2.9.0.

Analysis of filopodia growth speed was performed with Imaris 9.9.0 software. The tip of a growing filopodia was tracked using the spot function. The average growth speed of single filopodia was extracted from Imaris’ “statistics” function.

Filopodia were counted from acquired z-stacks using the multi-pointing tool from FIJI-ImageJ 2.9.0. 

### 2.5. Statistical Analysis 

Data were presented either as mean ± SEM or with median, and first and second quartile in violin plots. For multiple comparisons, one-way analysis of variance (ANOVA) with Tukey’s *t*-test was used. GraphPad Prism 9.3 was used for all statistical analysis and *p* values less than 0.05 were considered to indicate significant differences (* *p* < 0.05; ** *p* < 0.01; *** *p* < 0.001; **** *p* < 0.0001); *p* > 0.05, non-significant (NS). 

## 3. Results and Discussion

### 3.1. Efficient Filopodia Formation in MCF10A Cells Depends on FMNL2 and the Arp2/3 Complex

Different mechanisms have been proposed for the initiation and elongation of filopodia [30,31,32]. The convergent elongation model postulates that filopodial actin filaments assemble from an Arp2/3-dependent actin network [33]. The tip nucleation model postulates that nucleation and subsequent elongation are Arp2/3-independent and both are facilitated by formin proteins [32].

**Figure 1:**
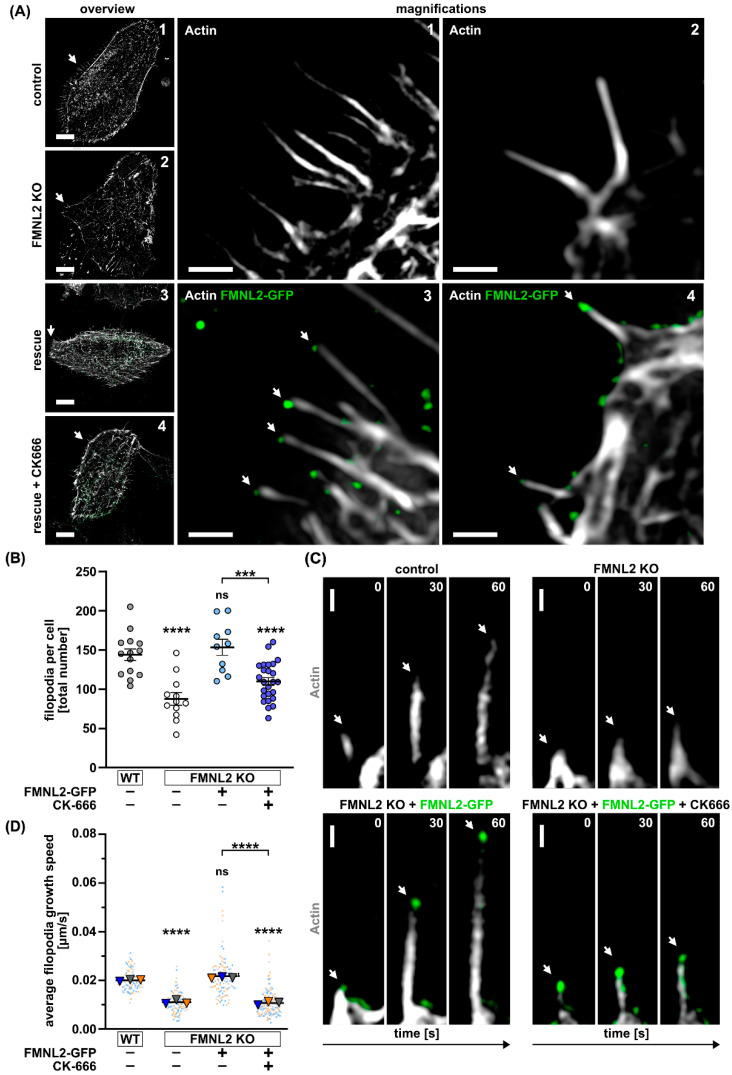
Filopodia formation in MCF10A cells depends on FMNL2 and Arp2/3 complex activity. Representative super-resolution live microscopy images of (**A**) MCF10A cells (Control = wildtype; FMNL2 KO; rescue = FMNL2 KO + FMNL2-GFP) transiently expressing actin-chromobody SNAP (white, SNAP-cell 647 SiR) and quantification of total filopodia per cell (**B**). Rescue cells were also treated with CK-666 (100 μM) for 30 min prior to imaging. White arrows in overview images (numbered from 1 to 4) indicate the location of magnified section with the corresponding number. Arrows in magnified images indicate FMNL2 (green) at the tip of filopodia (white). Scale bar (overview) = 10 μm. Scale bar (magnifications) = 1 μm. Data are shown as scatter plots with median (solid line) +/− SEM. Each spot represents an individual cell from at least three independent biological replicates. One-way ANOVA with Tukey’s multiple comparisons test was used for statistical analysis (*** = *p* < 0.001; **** = *p* < 0.0001; ns = not significant). Indicated drug treatments or expressions are labeled with (+). Absence of expression or treatment is labeled with (-). (**C**) Cells were subjected to super-resolution live-cell imaging with enhanced temporal resolution (burst mode, sliding processing) and the average filopodia growth speed was quantified (**D**). Filopodia growth for three different timepoints is shown. White arrows point towards growing filopodia. Scale bar = 500 nm. Graph shows the mean of biological replicates as a triangle. Dots in corresponding color to the triangle represent individual measurements of filopodia elongation rates. At least 10 cells were analyzed per condition. One-way ANOVA with Tukey’s multiple comparisons test was used for statistical analysis (**** = *p* < 0.0001; ns = not significant).

Since both models are not mutually exclusive and FMNL2 could have a role in both models, we wanted to analyze the contribution of FMNL2 to filopodia formation in MCF10A cells and also study the Arp2/3 dependency of filopodia formation. To this end, we used wild type (wt) cells, FMNL2 knock out (KO) cells and FMNL2 KO cells that were reconstituted by stable FMNL2-GFP expression (rescue) (Figure 1A). FMNL2 KO cells revealed a 39% reduction in filopodia formation compared to wt cells. Stable re-expression of FMNL2 could fully rescue filopodia formation and shows that FMNL2 is involved in the formation of a large fraction of filopodia in MCF10A cells (Figure 1B). Moreover, treatment with CK-666, an inhibitor of the Arp2/3 complex [34,35], reduced filopodia formation to the level of FMNL2 KO cells, suggesting that FMNL2 cooperates, at least to a significant degree, with Arp2/3 in filopodia elongation, and thus supporting a role for the convergent elongation model in our cell system. This is in line with a previous study which showed that the Arp2/3 complex and FMNL2 collaborate in actin assembly by promoting filament branching and subsequent filament elongation [11]. FMNL2’s main function in this context might be elongation rather than nucleation of actin filaments. New filament branches generated by Arp2/3 are captured and elongated by FMNL2.

To quantitatively analyze formin-dependent filopodia formation in more spatio-temporal detail, we measured the speed of filopodia elongation by structured illumination microscopy (SIM) with enhanced temporal resolution since FMNL2 should mediate the fast processive elongation of filopodia (Figure 1C,D). In FMNL2 KO cells, the elongation speed of filopodia was reduced by 45% and was fully rescued to wt levels by stable re-expression of FMNL2 (Figure 1D). Moreover, we could observe that FMNL-GFP accumulated at the tips of rapidly elongating filopodia in MCF10A rescue cells (Figure 1A,C and Video S1). Interestingly, treatment of FMNL2 rescue cells with CK-666 reduced the elongation speed of filopodia to the level of FMNL2 KO cells. Notably, the remaining filopodia were still positive for FMNL2-GFP (Figure 1C).

These data suggest that FMNL2 is involved in the fast elongation of filopodia. However, localization of FMNL2 to the filopodial tip is not sufficient and additional Arp2/3 activity is necessary for efficient elongation. One possibility could be that Arp2/3 activity contributes to an intact cellular actin cortex that may help facilitate efficient and rapid formin-mediated filopodia formation. Notably, it has been previously shown that Arp2/3 and formins cooperate in the assembly of the actin cortex [36], which may impact the formation of membrane protrusions. 

### 3.2. N-Myristoylation of FMNL2 Is Required for FMNL2-Dependent Filopodia Elongation

**Figure 2:**
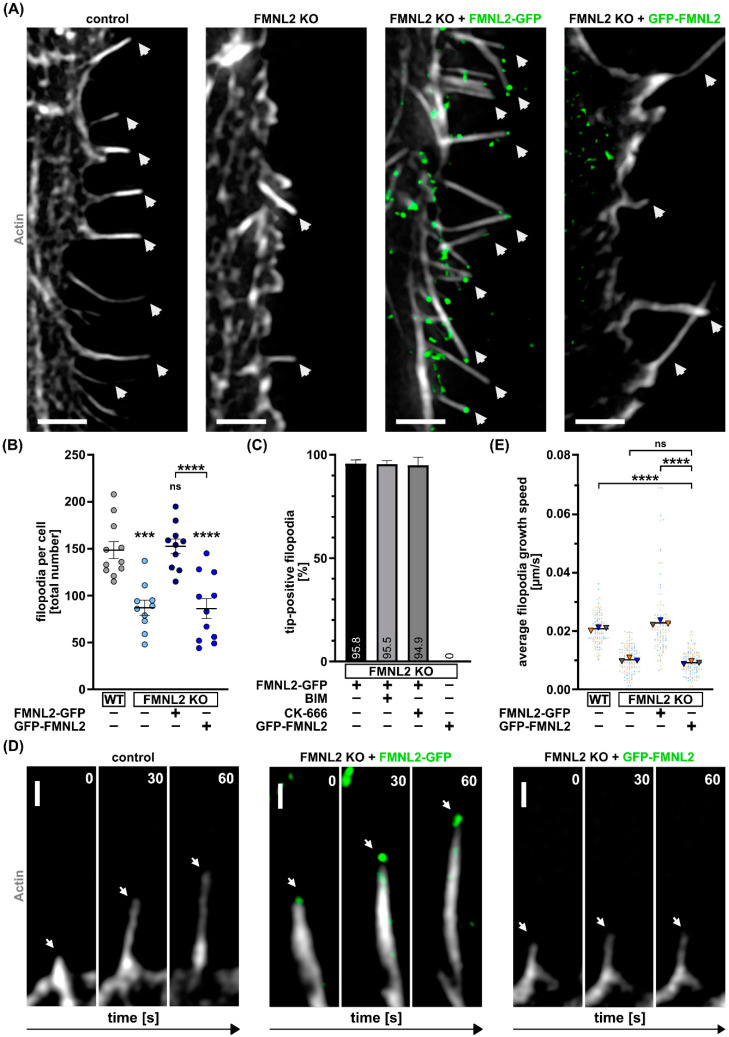
N-myristoylation of FMNL2 is required for FMNL2-dependent filopodia formation. Sections of MCF10A cells (Control = wildtype; FMNL2 KO; FMNL2 KO + FMNL2-GFP; green) exhibiting filopodia (**A**) and quantification of total number of filopodia per cell (**B**). Cells were transfected with actin-chromobody SNAP (white, SNAP-cell 647 SiR) and FMNL2 KO cells were additionally transfected with GFP-FMNL2 (green). White arrows indicate location of filopodia. Scale bar = 2 µm. Data are shown as scatter plots with median (solid line) +/− SEM. Each spot represents an individual cell from at least three independent biological replicates. For statistical analysis, one-way ANOVA with Tukey’s multiple comparisons test was used (*** = *p* < 0.001; **** = *p* < 0.0001; ns = not significant). Indicated drug treatments or expressions are labeled with (+). Absence of expression or treatment is labeled with (-). (**C**) Quantification of filopodia displaying FMNL2 at the tip in relation to the total number of filopodia per cell. Rescue cells expressing FMNL2-GFP (green) were treated with PKC inhibitor BIM (2 μM, 30 min) and Arp 2/3 complex inhibitor CK-666 (100 μM, 30 min) prior to imaging. FMNL2 KO cells were transiently transfected with GFP-FMNL2 (green). Bar charts show the mean percentage with SEM of FMNL2-positive filopodia from three independent biological replicates. At least 10 cells were analyzed per condition. (**D**) Representative time series and quantification (**E**) of the elongation rate of growing filopodia of MCF10A cells (Control = wildtype; FMNL2 KO; FMNL2 KO + FMNL2-GFP; green). Images show filopodia growth at three different timepoints (0 s, 30 s, 60 s). The white arrows point towards growing filopodia. Scale bar = 500 nm. Graph shows the mean of a biological replicate as a triangle. Dots in corresponding color to the triangle represent individual measurements of filopodia elongation rates. At least 10 cells were analyzed per condition. One-way ANOVA with Tukey’s multiple comparisons test was used for statistical analysis (**** = *p* < 0.0001; ns = not significant).

The FMNL2 protein is N-myristoylated at glycine 2 which targets it to the plasma membrane; while N-terminal tagging such as with GFP abrogates myristoylation, it does not affect GTPase interactions with the N-terminal formin GBD [11] or actin polymerization [13]. In previous studies, overexpression of C-terminally tagged FMNL2 increased the formation of filopodia, while overexpression of N-terminally tagged FMNL2 or a G to A mutation at position 2, which prevents N-myristoylation did not further increase the number of filopodia [13,26,28]. To address the role of N-myristoylation of FMNL2, we first analyzed the number of filopodia in our MCF10A FMNL2 KO cells in comparison to rescue cells expressing FMNL2 with an N- or C-terminal GFP-tag (Figure 2A). FMNL2 KO cells showed a 41% reduction of filopodia formation. In line with previous studies using overexpression in wt cells, re-expression of C-terminally tagged FMNL2 fully rescued filopodia formation in FMNL2 KO cells. In contrast, N-terminally tagged FMNL2 was not able to rescue filopodia formation and the number of filopodia was similar to the KO level (Figure 2B). This suggests that exclusively N-myristoylated FMNL2 is involved in filopodia formation. Accordingly, no filopodia tips with N-terminally tagged FMNL2 accumulations were observed while the majority of filopodia were positive for C-terminally tagged FMNL2 (Figure 2C).

We further analyzed the FMNL2-dependent elongation of filopodia in MCF10A FMNL2 KO cells (Figure 2D). As expected, re-expression of C-terminally tagged FMNL2 fully rescued filopodia elongation in terms of growth speed. In contrast, N-terminally tagged FMNL2 showed no rescue and the growth speed remained on the FMNL2 KO-level (Figure 2E). These data demonstrate that N-myristoylation, and hence plasma membrane anchoring, is crucial for efficient spatio-temporal assembly of filopodia by FMNL2. This further suggests a tight biochemical and lipid-based association of FMNL2 with the protruding plasma membrane, which may explain the specialized properties and efficiency of this particular formin to facilitate filopodia formation. 

### 3.3. FMNL2-Phosphorylation at Serine 1072 by PKCs Is Required for Efficient Filopodia Formation

**Figure 3:**
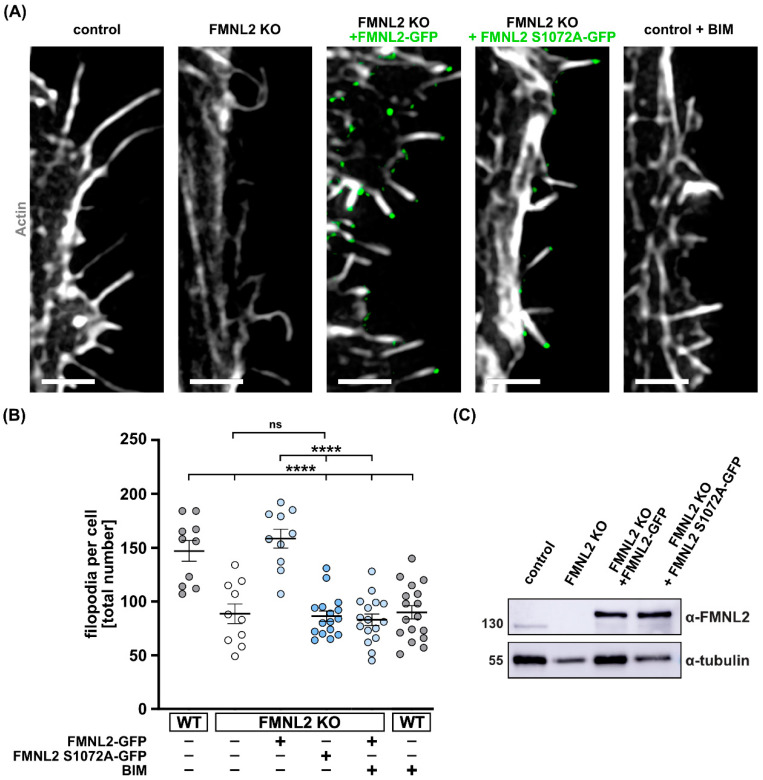
FMNL2-phosphorylation at S1072 by PKCs is involved in filopodia formation. Sections of MCF10A control (wildtype), FMNL2 KO and rescue cells (FMNL2 KO + FMNL2-GFP and FMNL2 KO + FMNL2 S1072A-GFP; green) exhibiting filopodia (**A**) and quantification of filopodia number per cell (**B**). Cells were transfected with actin-chromobody SNAP (white, SNAP-cell 647 SiR). Control cells and FMNL2 KO rescue cells were also treated with PKC inhibitor BIM (2 μM, 30 min). Scale bar = 2 μm. Data are shown as scatter plots with median (solid line) +/− SEM. Each spot represents an individual cell from at least three independent biological replicates. At least 10 cells were analyzed. For statistical analysis, one-way ANOVA with Tukey’s multiple comparisons test was used (**** = *p* < 0.0001; ns = not significant). Indicated drug treatments or expressions are labeled with (+). Absence of expression or treatment is labeled with (-). (**C**) Western blot analysis for FMNL2 of control, FMNL2 KO and rescue- cells.

Phosphorylation of formins to steer their activity has emerged as an important mechanism to regulate formin autoinhibition [37]. In our previous work, we identified FMNL2 as a substrate of PKCα [10,25]. A conserved serine at position 1072 within a RRSVR (R, Arg; S, Ser; V, Val) motif constitutes a target for classical PKCs. We have shown that phosphorylation at the respective serine residue releases FMNL2 from its autoinhibited state [10]. To analyze the impact of this phosphorylation on filopodia formation we stably re-expressed a mutant FMNL2 (S1072A) that cannot be phosphorylated by PKCs in FMNL2 KO cells (Figure 3A). Notably, the phosphorylation-deficient mutant was not able to rescue filopodia formation in MCF10A cells in contrast to wt FMNL2 (Figure 3B). Expression of the FMNL2 variants in stable cell lines was verified by Western blot (Figure 3C). Moreover, treatment of wt cells with a PKC inhibitor (BIM; Bisindolylmaleimide), selective for PKCα, β1, β2, γ, δ and ε isozymes, significantly reduced the formation of filopodia in wt cells. The number of filopodia was similar to FMNL2 KO cells after BIM treatment (Figure 3B). BIM treatment of rescue cells stably expressing FMNL2 reduced the number of filopodia back to the level of FMNL2 KO cells. Together, these results suggest that not only general PKC activity is necessary for FMNL2-mediated filopodia formation, but also phosphorylation of the specific serine residue at position 1072.

**Figure 4:**
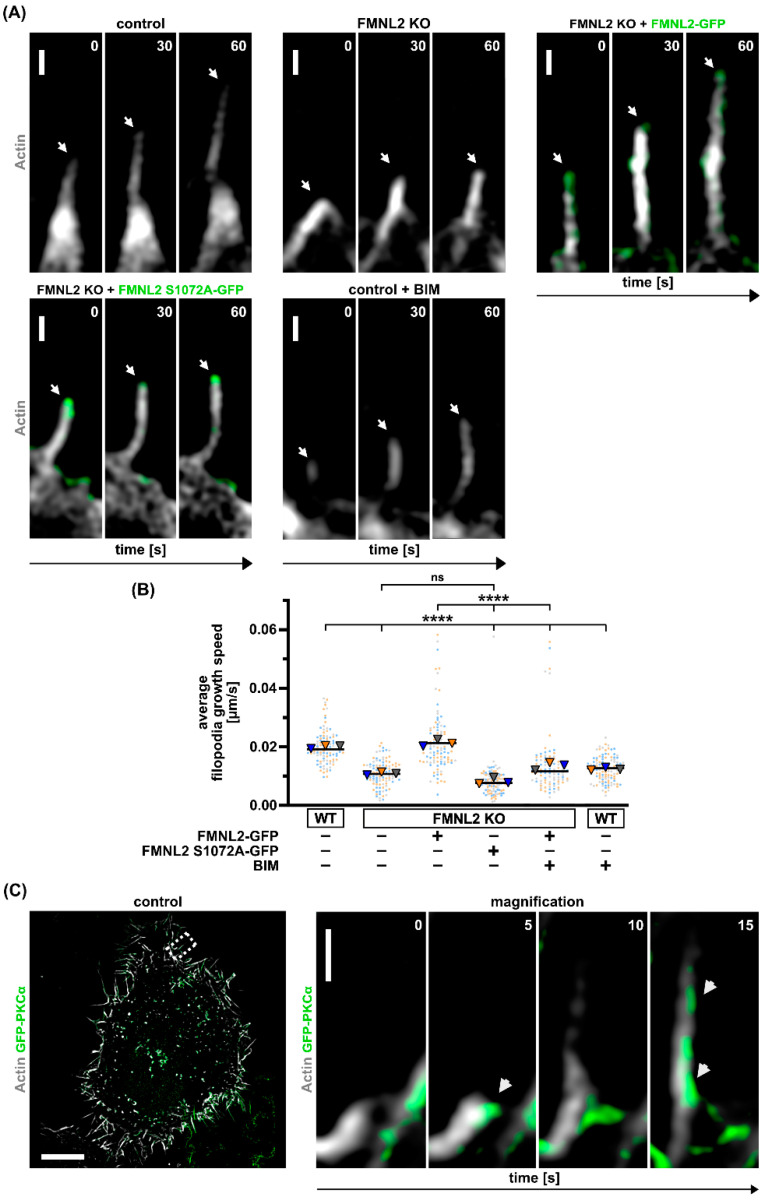
Inhibition of FMNL2 phosphorylation results in reduced filopodia growth speed in MCF10A cells. Representative images (**A**) and quantification (**B**) of average filopodia growth speed of MCF10A control (wildtype), FMNL2-KO and rescue cells (FMNL2-KO + FMNL2-GFP and FMNL2-KO + FMNL2 S1072A-GFP; green) that were transfected with actin-chromobody SNAP (white, SNAP-cell 647 SiR). Control cells were also treated with PKC inhibitor BIM (2 μM, 30 min). Images show time course of filopodia growth at three different timepoints. White arrows point towards the growing filopodia. Scale bar = 500 nm. Graph shows mean of a biological replicates as a triangle. Dots in corresponding color to the triangle represent individual measurements of filopodia elongation rates. At least 10 cells were analyzed per condition. One-way ANOVA with Tukey’s multiple comparisons test was used for statistical analysis (**** = *p* < 0.0001; ns = not significant). Indicated drug treatments or expressions are labeled with (+). Absence of expression or treatment is labeled with (-). (**C**) Control cells transiently expressing GFP-PKCα (green) and actin-chromobody SNAP (white, SNAP-cell 647 SiR). Left image shows an overview of a control cell. Dashed box indicates location of magnified region. Magnified region shows a time course of filopodia induction. White arrow indicates localization of PKCα at different timepoints during filopodia growth. Scale bar (overview) = 10 µm. Scale bar (magnification) = 500 nm.

Analysis of the filopodia growth speed in FMNL2 KO cells re-expressing wt FMNL2 or non-phosphorylatable FMNL2 (Figure 4A) revealed that non-phosphorylatable FMNL2 could not rescue filopodia growth speed in contrast to wt FMNL2. Growth speed remained similar to FMNL2 KO cells. Moreover, growth speed was significantly reduced in wt cells after treatment with the PKC inhibitor BIM (Figure 4B) and also in rescue cells stably expressing FMNL2. In both cell types, PKC inhibition reduced filopodia growth speed to the level of FMNL2 KO cells.

Interestingly, the localization of FMNL2 at filopodia tips was preserved for the non-phosphorylatable FMNL2. These results strongly suggest that the efficiency of filopodia elongation is dependent on the phosphorylation of FMNL2 and on PKC activity. Moreover, serine phosphorylation at position 1072 appears crucial for efficient elongation. We speculate that phosphorylation at this residue facilitates a sustained activation of FMNL2 and prevents a premature abortion of filopodia elongation by a return into the autoinhibited state. Our data further emphasize the critical importance of PKC-dependent phospho-regulation of FMNL2 activity. 

To further analyze the localization of PKCα responsible for FMNL2 phosphorylation, we transfected MCF10A wt cells with GFP-PKCα (Figure 4C). We observed that PKCα accumulates at the position where filopodia are initiated and subsequently elongated. This is consistent with the notion that FMNL2 activity is spatio-temporally controlled by PKCα. 

The role of PKCs in the process of filopodia formation seems to be ambivalent. On one hand, PKCs phosphorylate fascin at S39 [38]. Fascin is necessary for actin bundling in filopodia. Phosphorylation increases fascin mobility and at the same time, it reduces its ability to bundle actin filaments. A very fast turnover of fascin on actin filaments in filopodia was observed which might be evidence for repeated cycles of phosphorylation and dephosphorylation [15]. Thus, PKCs inactivate a protein necessary for filopodia formation. On the other hand, PKCs phosphorylate and activate FMNL2 and thereby increase filopodia formation. Interestingly, activated FMNL2 interacts with non-phosphorylated fascin [15]. Unfortunately, we have not identified the phosphatases involved in this process. However, it is tempting to speculate that PKCs liberate fascin from F-actin, which is subsequently dephosphorylated and dragged by activated FMNL2 into nascent filopodia to stabilize them.

Moreover, the role of PKCs seems to vary in different cell types and for the respective isoforms. In platelets, it was shown that PKC activation decreases the number of filopodia [39]. On the other hand, in vascular endothelial cells, PKCs stimulate the formation of actin stress fibers and filopodia [40]. An early study in MCF10A suggested that overexpression of PKCα changed the cellular morphology to a non-aggregated and irregularly shaped phenotype with prominent lamellipodia and filopodia [41]. 

## 4. Conclusions

**Figure 5:**
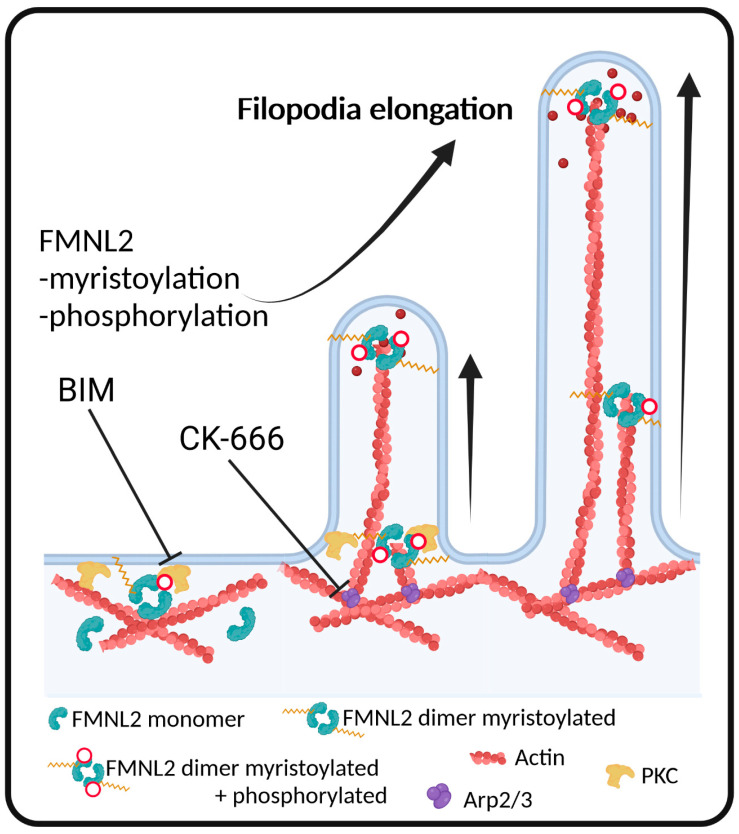
Model of FMNL-phosphorylation-dependent filopodia formation. Filopodia are assembled at the plasma membrane where actin, FMNL2 dimers and PKCs are present. N-terminal myristoylation enables plasma membrane anchoring of FMNL2. The formin is released from its autoinhibited state through phosphorylation by PKCα. Following Arp2/3 complex-mediated nucleation of actin filaments, myristoylated and phosphorylated FMNL2 localizes at the filopodial tip and further elongates the filopodia. Inhibition of the Arp2/3 complex with CK-666 or PKC activity with BIM reduces the overall filopodia growth rate and formation. Figure was created using BioRender.com.

We showed that phosphorylation of FMNL2 at serine 1072 increases the formation of filopodia. These filopodia can further enhance cell motility, migration and invasion as observed in several studies downstream of PKCα signaling in cancer and non-cancerous cells [10,23,42,43,44,45,46]. FMNL2-dependent filopodia formation is regulated on multiple levels. The filopodia localization of FMNL2 is independent of the phosphorylation state and N-myristoylation appears to be sufficient to recruit FMNL2 to filopodial tips (Figure 5). Furthermore, FMNL2-dependent filopodia formation requires Arp2/3 activity. This suggests that FMNL2-dependent filopodia are formed according to the convergent elongation model which postulates that filopodial actin filaments assemble from an Arp2/3-dependent cortical actin network [33]. Our data also uncover a functional requirement of PKCα for FMNL2 phosphorylation at serine 1072 and subsequent formin-mediated filopodia formation. Thus, myristoylation, Arp2/3 activity and serine phosphorylation cooperate to spatially and temporally control FMNL2 function for membrane protrusive actin assembly.

## Data Availability

The data presented in this study are available on request from the corresponding author.

## Supplementary Materials

The following supporting information can be downloaded at: https://www.mdpi.com/article/10.3390/biom13030548/s1, Video S1: MCF10A FMNL2 KO rescue cells stably re-expressing FMNL2-GFP display FMNL2 at the tip of growing filopodia. Cells were transfected with actin-chromobody SNAP (white, SNAP-cell 647 SiR). Scale bar = 500 nm.
